# Interspecies Variation in NCMN*-O-*Demethylation in Liver Microsomes from Various Species

**DOI:** 10.3390/molecules24152765

**Published:** 2019-07-30

**Authors:** Ziru Dai, Guibo Sun, Jiada Yang, Jie Hou, Ping Zhou, Weijie Xie, Guangbo Ge, Xiaobo Sun, Ling Yang

**Affiliations:** 1 Key Laboratory of Bioactive Substances and Resources Utilization of Chinese Herbal Medicine, Ministry of Education, Institute of Medicinal Plant Development, Peking Union Medical College and Chinese Academy of Medical Sciences, Beijing 100193, China; 2 School of Environment and Life Science, Kaili University, Kaili 556011, China; 3 College of Basic Medical Sciences, Dalian Medical University, Dalian 116044, China; 4 Institute of Interdisciplinary Integrative Medicine Research, Shanghai University of Traditional Chinese Medicine, Shanghai 201203, China

**Keywords:** NCMN*-O-*demethylation, Cytochrome P450 1A (CYP1A), species differences, liver microsomes

## Abstract

NCMN (*N*-(3-carboxy propyl)-4-methoxy-1,8-naphthalimide), a newly developed ratiometric two-photon fluorescent probe for human Cytochrome P450 1A (CYP1A), shows the best combination of specificity and reactivity for real-time detection of the enzymatic activities of CYP1A in complex biological systems. This study aimed to investigate the interspecies variation in NCMN*-O-*demethylation in commercially available liver microsomes from human, mouse, rat, beagle dog, minipig and cynomolgus monkey. Metabolite profiling demonstrated that NCMN could be *O*-demethylated in liver microsomes from all species but the reaction rate varied considerably. CYP1A was the major isoform involved in NCMN*-O-*demethylation in all examined liver microsomes based on the chemical inhibition assays. Furafylline, a specific inhibitor of mammalian CYP1A, displayed differential inhibitory effects on NCMN*-O-*demethylation in all tested species. Kinetic analyses demonstrated that NCMN*-O-*demethylation in liver microsomes form rat, minipig and cynomolgus monkey followed biphasic kinetics, while in liver microsomes form human, mouse and beagle dog obeyed Michaelis-Menten kinetics, the kinetic parameters from various species are much varied, while NCMN*-O-*demethylation in MLM exhibited the highest similarity of specificity, kinetic behavior and intrinsic clearance as that in HLM. These findings will be very helpful for the rational use of NCMN as a practical tool to decipher the functions of mammalian CYP1A or to study CYP1A associated drug-drug interactions *in vivo*.

## 1. Introduction

Cytochrome P450s (CYPs) are a superfamily of multifunctional hemoprotein monooxygenases that play an indispensable physiological role in the oxidative biotransformation of endogenous and xenobiotic compounds [1,2]. Among all known CYP enzymes, the CYP1A subfamily has been amply studied due to they contribute most extensively to the bioactivation of procarcinogen such as aflatoxins, polycyclic aromatic hydrocarbons and heterocyclic arylamines, which are conductive to tumor formation and cancer susceptibilities [3,4,5]. In most mammals, the CYP1A subfamily comprises of two isozymes (CYP1A1 and CYP1A2). These two isoforms share high homology in amino acid sequence and overlapping profiles of substrate. Generally, CYP1A2 is primarily expressed in the liver (~13–15%), while CYP1A1 expression levels are relatively low (<0.7%), it is mostly distributed in extrahepatic tissues [6,7,8]. Notably, the function and expression of CYP1A can be regulated by a wide range of environmental factors (such as smoking, high-level aflatoxin exposure) and therapeutic drugs [5,9,10]. Dysfunction or abnormal expression of CYP1A may trigger some serious undesirable effects, owing to CYP1As (including both CYP1A1 and CYP1A2) are key enzymes participating in the metabolic activation of procarcinogens to reactive intermediates or ultimate carcinogens inducing subsequent mutagenesis and tumorigenesis, which significantly correlates with the susceptibilities to lung and liver cancers [11,12]. 

**Figure 1:**
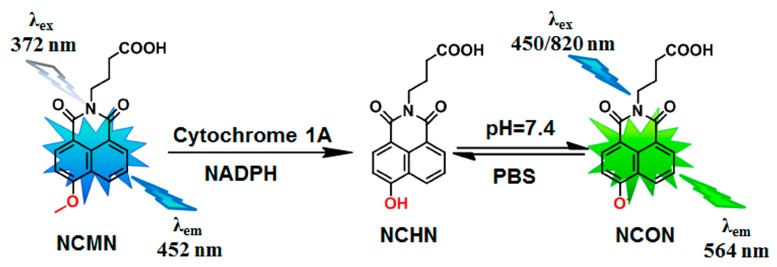
The chemical structure of *N*-(3-carboxy propyl)-4-methoxy-1,8-naphthalimide (NCMN) and its fluorescence response towards mammalian cytochromes 1A from different species.

It is generally believed that selecting a suitable animal model(s) is very crucial for pharmacological and toxicological related investigations of a new drug candidate [13,14]. Considering that CYP1A enzymes participate in the metabolic clearances of a wide variety of xenobiotics (such as therapeutic drugs and food chemicals) and in the metabolic activation of many procarcinogens to endotoxic intermediates, it is necessary to decipher the real functions of mammalian CYP1A in living systems using some practical tools, such as optical probe substrates [15,16,17,18,19,20,21]. NCMN (*N*-(3-carboxy propyl)-4-methoxy-1,8-naphthalimide) is a newly developed two-photon ratiometric fluorescent probe for mammalian CYP1A, which has been favorably utilized for real-time monitoring CYP1A in living cells and tissues [22]. Compared with traditionally used probe substrates for CYP1A (such as caffeine and phenacetin), NCMN exhibits excellent performance and many advantages, such as high selectivity, deep tissue penetration, as well as strong anti-interference ability in complex biological systems [23,24]. In addition, because of its low cytotoxicity and deep-tissue imaging depth, NCMN could be successful applied to visualize endogenous CYP1A in living cells and tissues. The superiorities of NCMN provide a promising tool for in-situ monitoring the real activities of CYP1A in living systems (Figure 1). Notably, although the specificity and the response of NCMN towards human CYP1A have been well investigated, the specificity and the capability of CYP1A for sensing mammalian CYP1A have not been well studied [22]. Thus, it is necessary to ascertain whether NCMN is also a specific probe substrate for CYP1A in other animal species, especially for those commonly used experimental animals.

The scope of this study is to investigate the interspecies variations on NCMN*-O-*dealkylation in liver preparations of various animal species. To this end, the commercially available liver microsomes (such as pooled human liver microsomes, as well as liver microsomes form mouse, rat, beagle dog, minipig and cynomolgus monkey) were collected, while the interspecies variations on NCMN*-O-*dealkylation in these liver preparations were carefully investigated by a panel of experimental animal models, including metabolite profiling, enzymatic kinetics analyses and inhibition assays. All these findings are very helpful for exploring the specificity and the sensing capability of NCMN*-O-*dealkylation towards CYP1A in commonly used experimental animals, which will be very useful for further investigations on CYP1A-asscoaited biological events using NCMN as a specific tool for sensing CYP1A activities in animals.

## 2. Results

### 2.1. NCMN-O-Dealkylation in Liver Microsomes from Various Species

**Figure 2:**
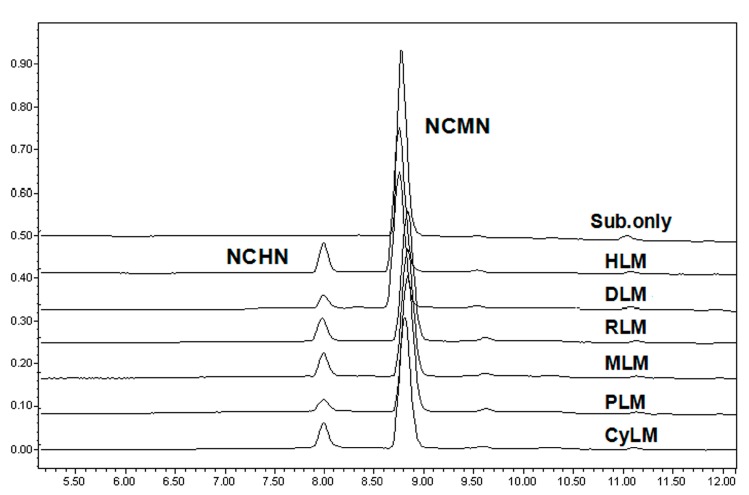
Representative LC-UV profiles of NCMN and its demethylation metabolite in liver microsomes from human, beagle dog, rat, mouse, minipig and cynomolgus monkey. NCMN (10 µM) was incubated with liver microsomes (0.25 mg protein/mL) from each species at 37 °C for 30 min.

The metabolite profiles of NCMN in liver microsomes from different animal species were depicted following 30 min co-incubation with liver microsomes from mouse, rat, minipig, beagle dog, cynomolgus monkey and human (0.25mg protein/mL), in the presence of the NADPH-generating system (Figure 1). As shown in Figure 1, NCMN could be readily O-demethylated in all examined liver microsomes from mammals. A single metabolite of NCMN was fully detected and identified as NCHN (N-(3-carboxy propyl)-4-hydroxy-1,8-naphthalimide), by comparing the retention times, Ultraviolet (UV) and mass spectrometer (MS) spectra with the standard (supplementary Figure S1). The formation of these metabolites was NADPH-, time-and microsomes-dependent. The formation rates of NCHN in liver microsomes from different species were seem to be similar with that of human liver microsomes, whearas HLM showed the fastest O-dealkylation rate of NCMN among all examined hepatic microsomes (Figure 2).

### 2.2. Inhibitory Effects of CYP450 Inhibitors on NCMN-O-Dealkylation

**Figure 3:**
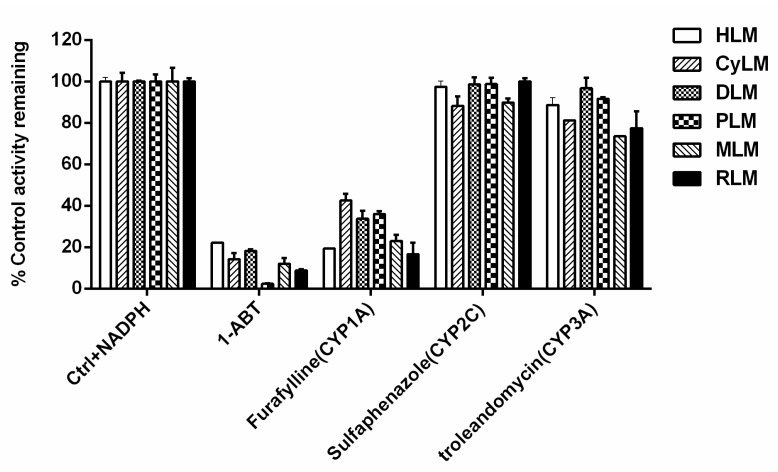
The inhibitory effects of chemical inhibitors of cytochrome P450s (CYPs) on the formation of NCMN in liver microsomes from different species. N.D. means not detected.

A series of CYP-specific inhibitors were used to investigate the involved enzymes responsible for NCMN*-O-*dealkylation in different species. As shown in Figure 3, ABT (a broad specificity CYP inhibitor) could potently inhibit the formation of NCHN, suggesting that NCMN O-dealkylation was predominantly mediated by mammalian P450 enzymes in the liver from different animal species. After that, some known selective inhibitors of mammalian CYP isoforms were used to explore the major CYP isoform participating in NCMN*-O-*dealkylation. As shown in Figure 3, furafylline, a potent selective inhibitor for mammalian CYP1A, exhibited different inhibitory effects on the formation of NCHN, which inhibited NCMN*-O-*dealkylation near completely in human, rat and mouse, while lower inhibitory effects have been observed in cynomolgus monkey, beagle dog and minipig. Moreover, sulfaphenazole (a selective inhibitor of mammalian CYP2C) and troleandomycin (a selective inhibitor of mammalian CYP3A), weakly inhibited the formation of NCHN (less than 25% inhibition, *p* > 0.05), in all examined species. These results clearly demonstrated that NCMN*-O-*demethylation was mainly catalyzed by mammalian CYP1A in liver preparations of all tested species.

### 2.3. Inhibition of Furafylline on NCMN-O-Dealkylation in Liver Microsomes from Various Species

**Figure 4:**
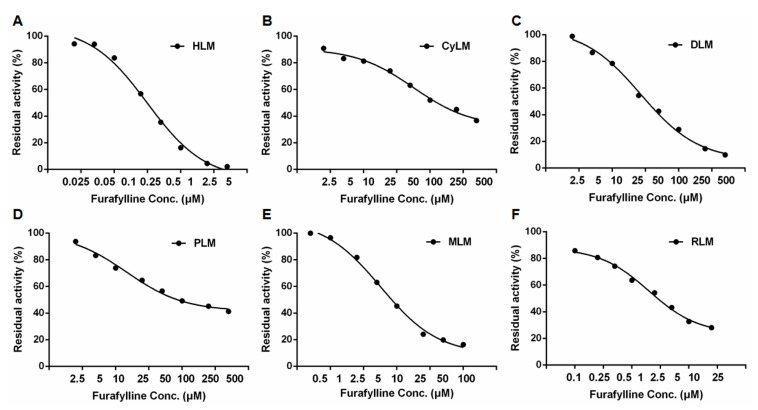
The dose-dependent inhibition curves of furafylline on NCMN*-O-*demethylation in liver microsomes from human (**A**), cynomolgus monkey (**B**), beagle dog (**C**), minipig (**D**), mouse (**E**) and rat (**F**).

In addition to investigate the similarities of NCMN*-O-*dealkylation in liver microsomes from different species, the dose-dependent inhibition curves of furafylline (a known CYP1A inhibtor) on NCMN*-O-*dealkylation were also investigated using varying inhibitor concentrations, as depicted in Figure 4 and Table 1. The 50% inhibition concentration (IC_50_) values of furafylline agaisnt NCMN*-O-*dealkylation in cynomolgus monkey liver microsomes (CyLM), beagle dog liver microsomes (DLM), minipig liver microsomes (PLM), mouse liver microsomes (MLM) and rat liver microsomes (RLM) were then evaluated as 56.36 µM, 26.77 µM, 14.61 µM, 5.73 µM and 1.98 µM, which is 6.4 fold (RLM) to 181.8 fold (CyLM) higher than that observed with HLM (IC_50_ = 0.31 µM). The significant differences in inhibitory effects of furafylline towards mammalian CYP1A suggested that furafylline is more sensitive to human CYP1A mediated NCMN*-O-*dealkylation, but this chemical inhibitor is insensitive towards CYP1A in other mammals. These results demonstrated that furafylline could be a more selective and sensitive inhibitor against CYP1A-mediated NCMN*-O-*demethylation in human rather than mammalian CYPs from above mentioned animal species.

### 2.4. Kinetic Analyses of NCMN-O-Demethylation in Liver Microsomes from Various Species

**Figure 5:**
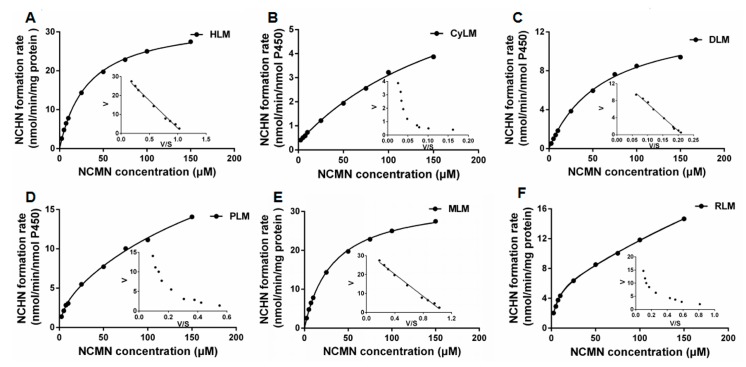
Enzymatic kinetic plots of NCMN*-O-*demethylation in liver microsomes from human (**A**), cynomolgus monkey (**B**), beagle dog (**C**), minipig (**D**), mouse (**E**) and rat (**F**). NCMN (1–150 µM) was incubated with HLM, CyLM, DLM, PLM, MLM or RLM(F) at 37 °C. Please add explanations for (A)–(F)

The comparative kinetic analyses for NCMN*-O-*demethylation in liver microsomes from different species were also performed. NCMN*-O-*demethylation in liver microsomes from human, beagle dog and mouse, revealed Michaelis-Menten profile, as evidenced by Eadie-Hofstee plot (Figure 5). However, NCMN*-O-*demethylation in liver microsomes from rat, cynomolgus monkey and minipig, obeyed the biphasic kinetics (Figure 5). As shown in Table 2, the estimated *K_m_* values for NCHM formation ranged widely in liver microsomes from above species, from 0.18 to 1134 µM, while the *V_max_* values range in NCHM formation was from 0.28 to 78.42 nmol/min/mg protein. As a result, the intrinsic clearance values for the formation of NCHM varied remarkably among different species, ranging from 218.96 mL/min/mg liver microsomes to 2684.48 mL/min/mg liver microsomes. Moreover, the enzymatic kinetic of NCMN*-O-*demethylation in rat recombinant CYP1A2 were also investigated, which obeyed Michaelis-Menten kinetics, displayed similar *K_m_* values and *V_max_* values with that in RLM (supplementary Figure S2 and Table S1). The order of the intrinsic clearance for the formation of NCHM as follows, Human > Cynomolgus Monkey > Mouse > Rat > Minipig > Beagle Dog, which were in accordance with the tendency of the O-demethylation profiles of NCMN in the above mentioned enzyme sources.

## 3. Discussion

It is well-known that human CYP1A enzymes (including CYP1A1 and CYP1A2) are key enzymes participating in bioactivation of many procarcinogens to endotoxic intermediates, and are thereby involved in the process of carcinogenesis [25]. It also has been reported that CYP1A1 can be induced by a variety of environmental pollutants and then converts them to carcinogenic metabolites, while the elevated CYP1A1 activity is believed to associate with a high risk of various cancers, such as lung cancer and colorectal cancer [26]. Meanwhile, CYP1A2 can also metabolically activate some known procarcinogens to the corresponding carcinogens, and thus has a significant effect on human tobacco-related cancers [27,28]. Currently, CYP1A has been recognized as a promising target for the prevention of chemical carcinogenesis. However, the species difference or the similarity in the function of mammalian CYP1A enzymes, as well as their responses towards CYP1A inhibitor(s) have not been well investigated. Considering that CYP1A plays crucial roles in metabolic clearance of a wide variety of drugs or food chemicals, as well as in the bioactivation of many procarcinogens, it is necessary to investigate the species difference or the similarity in the function of CYP1A enzymes from various mammals. Such investigation will strongly facilitate to select an appropriate laboratory animal(s) with respect to the similar tissue distribution, metabolic behaviors and biological responses of CYP1A in human, which will be very helpful for *in vivo* studies of CYP1A substrate drugs or procarcinogens.

Recently, NCMN (*N*-(3-carboxy propyl)-4-methoxy-1,8-naphthalimide), a novel ratiometric two-photon fluorescent probe for sensing human cytochrome P450 1A (CYP1A), has been developed by us for real-time detection of the enzymatic activities of CYP1A in complex biological systems [22]. Although this newly developed fluorogenic probe substrate shows high specificity, low cytotoxicity and good reactivity for sensing the enzymatic activities of human CYP1A in complex biological system, the interspecies difference in NCMN*-O-*dealkylation, especially for the specificity and the capability of this probe reaction for sensing mammalian CYP1A have not been well studied. In the present study, to explore the sensitivity and applicability of NCMN for sensing mammalian CYP1A from commonly used experimental animals, the interspecies variation in NCMN*-O-*dealkylation was thoroughly studied using liver microsomes from various species interspecies, including human, mouse, rat, beagle dog, minipig and cynomolgus monkey.

Metabolite profiling of NCMN demonstrated that NCMN*-O-*demethylation could be rapidly catalyzed in liver microsomes from all examined species (Figure 2). Chemical inhibition assays suggested that furafylline (a selective inhibitor against CYP1A) showed moderate to strong inhibition of NCMN*-O-*demethylation in all tested species, suggesting that the NCMN*-O-*demethylation in above mentioned species was mainly mediated by mammalian CYP1A (Figure 3). Enzymatic kinetic analyses demonstrated that the kinetic behaviors of NCMN*-O-*demethylation are much varied in liver microsomes from various species, and none of the selected animal species is totally parallel to humans in terms of kinetic parameters. NCMN*-O-*demethylation in RLM, PLM and CyLM followed biphasic kinetic, which could be explained by that two CYP1A enzymes (including CYP1A1 and CYP1A2) in the liver of these species are two contributors involved in NCMN O-demethylation, owing to CYP1A1 and CYP1A2 are expressed at similar levels in liver microsomes of rat, minipig and cynomolgus monkey [29,30]. Notably, NCMN*-O-*demethylation in rat CYP1A2 followed Michaelis-Menten kinetic, which is different from this reaction in RLM (biphasic kinetic), suggesting that both rat CYP1A1 and CYP1A2 were involved in NCMN O-demethylation in RLM. The *K_m_* value of NCMN*-O-*demethylation in rat CYP1A2 is similar to the lower *K_m_* value of NCMN*-O-*demethylation in RLM, suggesting that NCMN may bind on CYP1A2 with high affinity in contrasat to CYP1A1. By comparison, NCMN*-O-*demethylation in HLM, MLM and DLM obeyed classic Michaelis-Menten kinetics, as CYP1A2 is copiously expressed in the liver, while CYP1A1 is expressed only at very low levels in the liver of human, mouse and beagle dog [31,32]. The abundant expression of CYP1A2 in human liver also explained why the enzymatic kinetics plots and kinetic parameters of NCMN*-O-*demethylation in HLM are much closed to that in CYP1A2 (supplementary Table S1). Notably, the affinities of NCMN towards mammalian CYP1A from various species are much varied to each other, from 9.54 µM (in HLM) to 1134.00 µM (in RLM). Furthermore, the intrinsic clearance values (*V_max_*/*K_m_*) for NCMN in HLM are much different from that in DLM or PLM, implying that a beagle dog or minipig could not serve as a good surrogate model to decipher the real function of CYP1A using NCMN*-O-*demethylation as a probe reaction, which was in accordance with a previous report about CYP1A [33]. Although the amino acid sequence identity between human CYP1A and mammalian CYP1A enzymes were very high (supplementary Figure S3 and Tables S2 and S3), the large difference in NCMN*-O-*demethylation in liver preparations from various mammals suggested that some key amino acids of mammalian CYP1A might play important roles in the recognition, binding and catalysis of NCMN. It has been reported that the active site residues Arg107, Ser122, Thr123, Thr124, Thr126, Asp313, Gly314, Gly316, Ala317, Gly320, Thr321, Leu448, Arg454, Cys456, Thr498, and Thr501 in CYP1A1 and CYP1A2 enzymes across human, rat and mouse are involved in binding with the ligands, when these residues formed different interactions with ligands, species difference in the recognition, binding and catalysis of ligands might be observed for CYP1A enzymes [34]. By comparison, similar kinetic behaviors of NCMN*-O-*demethylation in MLM were observed as that in HLM. Notably, the *K_m_*, *V_max_* as well as intrinsic clearance values for the formation of NCHN in MLM are close to that in HLM. These findings suggested that CYP1A was the major enzyme responsible for NCMN*-O-*demethylation in all species tested, while MLM exhibited the best combination of specificity, kinetic behavior and intrinsic clearance as that in HLM.

Over the past two decades, increasing attention has been focused on the biological consequences of CYP1A inhibitors. On one hand, inhibition of these enzymes has been recognized as one of the most promising therapeutic strategies against cancer, since CYP1A plays a crucial role in the formation of reactive intermediates leading to mutagenesis and tumorigenesis [35,36,37]. On the other hand, inhibition on CYP1A may also affect the metabolism and detoxification of a wide range of xenobiotics, thereby modulating the half-lives, bioavailability and the therapy effects of CYP1A substrates [38]. For instance, co-administration of enoxacin (a quinolone antibiotic), which is capable of inhibiting CYP1A *in vivo*, can lead to reduction of the metabolic clearance of R-warfarin (a CYP1A substrate drug), and thereby bring undesirable effects [39,40]. In the present study, furafylline, a well-known inhibitor of CYP1A [41], has been selected as a positive inhibitor of CYP1A, to explore the similarity of NCMN*-O-*demethylation in liver microsomes from all studied species. It was obvious from Figure 4 and Table 1 that a high dosage of furafylline (500 μM, final concentration) could significantly inhibit mammalian CYP1A mediated NCMN*-O-*demethylation, but the IC_50_ values varied considerably (0.31–56.36 μM). These findings demonstrated that mammalian CYP1A from all examined animals appeared to be less sensitive towards furafylline CYP1A in comparison with human CYP1A, which were in agreement with previous studies [28,42]. Notably, similar results have been reported that furafylline also displayed relative weak inhibition on 7-ethoxyresorufin O-deethylation (a probe substrate for CYP1A) from mouse, rat, cynomolgus monkey and beagle dog when compared with human CYP1A. Taken together these results indicated obvious species-differences in sensitivity to CYP1A inhibitors, which may not only contribute to the major differences in the active site geometry between human and mammalian orthologues of CYP1A, but might also be due to the differences in the competing pathways or the pharmacokinetics of the inhibitors [14]. These findings suggested that high dosage of CYP1A inhibitors should be used to study CYP1A-associated biological processes in these animals.

## 4. Materials and Methods

### 4.1. Chemicals and Reagents

*N*-(3-carboxy propyl)-4-methoxy-1,8-naphthalimide (NCMN) and *N*-(3-carboxy propyl)-4-hydroxy-1,8-naphthalimide (NCHN) were synthesized by our laboratory with purity above 98% identified by HPLC-UV. 1-Aminobenzotriazole (ABT), sulfaphenazole, troleandomycin, furafylline, glucose-6-phosphate dehydrogenase, NADP+ and d-glucose-6-phosphate were purchased from Sigma (St. Louis, MO, USA). All other reagents were reagent grade or better. Pooled human liver microsomes (HLMs, from 50 donors, lot no. X008067) were obtained from Bioreclamation IVT (Baltimore, MD, USA). Liver microsomes form male ICR/CD-1 mouse (Lot. STOM, *n* = 500), male Sprague-Dawley rat (Lot.JPXY, *n* = 100), male beagle dog (Lot.DMXD, *n* = 3), male Yucatan minipig (Lot.GGSK, *n* = 2), male cynomolgus monkey (Lot.CYJC, *n* = 3) as well as cDNA-expressed rat CYP1A2 (Lot.int066e1c) were purchased from Research Institute for Liver Diseases (RILD, Shanghai, China). 

### 4.2. Incubation Conditions

The 200 µL incubates contained 0.125 to 0.25 mg protein/mL liver microsomes or 100 nM CYPs, an NADPH-generating system (1 mM NADP+, 10 mM glucose-6-phosphate, 1 unit/m glucose-6-phosphate dehydrogenase, and 4 mM MgCl_2_) in 100 mM potassium phosphate buffer (pH 7.4). After pre-incubation at 37 °C for 3min, the incubation was commenced by addition of NADPH-generating system, maintained at 37 °C. The reaction was quenched with ice-cold acetonitrile (200 µL). The mixture was chilled, spun at 20,000× *g* for 20 min at 4 °C. All incubations throughout the study were done in three experiments.

### 4.3. Kinetic Analyses

The formation rates of NCHN were within the linear range of the protein concentration and incubation time. NCMN (1, 2.5, 5, 7.5, 10, 25, 50, 75, 100 and 150 µM) was incubated with HLM, MLM, RLM, DLM, PLM, CyLM (0.25mg protein/mL) as well as rat CYP1A2 (100 nM) for 30min. The apparent *K_m_* and *V_max_* values were determined by fitting the substrate concentrations versus the metabolite reaction velocities to the Michaelis-Menten equation (Equation (1)) and the Biphasic Kinetics equation (Equation (2)), and the results were graphically represented by Eadie-Hofstee plots.

(1) $$v=\frac{V_{\max}[S]}{K_{m}+[S]}$$

(2) $$v=\frac{V_{\max\;1}[S]}{K_{\max\;1}+[S]}+\frac{V_{\max\;2}[S]}{K_{\max\;2}+[S]}$$

where *v* is the rate of the reaction, *V_max_* is the maximum velocity estimate, *K_m_* is the substrate affinity constant, [*S*] is the substrate concentration.

### 4.4. Chemical Inhibition Assays

Furafylline, sulfaphenazole, troleandomycin and 1-ABT were used to inhibit NCMN O-demethylation in HLM, MLM, RLM, DLM, PLM and CyLM. 10 µM NCMN was incubated with an NADPH-generating system with or without CYP inhibitors: 100 µM furafylline (a CYP1A inhibitor), 10 µM sulfaphenazole (a CYP2C inhibitor), 200 µM troleandomycin (a CYP3A inhibitor) and 500 µM 1-ABT (a broad CYPs inhibitor) [43,44,45,46,47]. Moreover, the inhibitory effects and the IC_50_ values of furafylline (0–500 μM) were investigated using various concentrations of NCMN in liver microsomes from mammals. 

### 4.5. Fluorescence Detection and LC-ESI-MS Analysis

All fluorescence studies for NCMN and and its O-demethylation metabolite (NCHN) were carried out on a multi-mode microplate reader (BioTek Synergy H1, Hybrid Reader, Winooski, VT, USA), under the fluorescence emission of 452 nm and 564 nm and the fluorescence excitation of 372 nm and 450 nm, respectively (Gain = 60 for substrate and Gain =100 for metabolite).

The UFLC system consisted of a SIL-20ACHT auto sampler, a DGU-20A3 in-line degasser, a CBM-20A communications bus module, a CTO-20AC column oven, two LC-20AD pumps and an SPD-M20A photodiode array detector were used for analyzing the samples. The separation of NCMN and its metabolites was achieved on a Shim-pack XR-ODS (75 mm × 2.0 mm, 2.2 µm, Shimadzu, Kyoto, Japan) analytical column with an ODS guard column (5 mm × 2.0 mm, 2.2 µm, Shimadzu, Kyoto, Japan). The O-demethylation was quantified at the detector wavelength of 243 nm. The mobile phase was comprised of CH_3_CN (A) and 0.2% formic acid (B) at total flow rate of 0.4 mL/min, which was linear from 90% B to 80% B in 2 min, then decreased to 25% B in 8 min, followed by a decrease to 5%B in 4 min, and finally balanced to 90% B in 4 min. The column oven was kept at 40 °C. 

NCMN and NCHN were identified by means of a Shimadzu LC-MS-2010EV (Kyoto, Japan) instrument with an ESI interface. In respect to mass detection, positive-ion mode (ESI+) as well as negative ion mode (ESI−) from *m*/*z* 100 to 800 with the electron voltage setting at +1.55 kV, and −1.55 kV was employed. Data processing was conducted using the LC-MS Solution (version 3.41; Shimadzu, Kyoto, Japan).

## 5. Conclusions

In summary, the interspecies difference in NCMN*-O-*demethylation was well-characterized in liver preparations of various species, in regard to the similarities in metabolic profiles, enzymatic kinetics analyses and inhibition assays. The results clearly demonstrated that mammalian CYP1A were the primary enzymes responsible for NCMN*-O-*demethylation in liver microsomes from human and five studied animals. Further investigation demonstrated that NCMN*-O-*demethylation in MLM exhibited the best combination of specificity, kinetic behaviors as well as intrinsic clearances as that in HLM. In addition, the inhibitory effects of furafylline on NCMN*-O-*demethylation varied considerately among different animals. All these findings were helpful for the further investigation on CYP1A-associated physiological and pathological processes using NCMN*-O-*demethylation as a marker reaction to decipher the real functions of mammalian CYP1A, as well as for *in vivo* studies on CYP1A-mediated drug-drug interactions.

## Figures and Tables

**Table 1:** The IC_50_ values of furafylline against NCMN*-O-*demethylation in liver microsomes from different species.

Enzyme Source	IC_50_ (µM)
HLM	0.31
CyLM	56.36
DLM	26.77
PLM	14.61
MLM	5.73
RLM	1.98

Human liver microsomes (HLM), cynomolgus monkey liver microsomes (CyLM), beagle dog liver microsomes (DLM), minipig liver microsomes (PLM), mouse liver microsomes (MLM) and rat liver microsomes (RLM).

**Table 2:** Kinetic parameters of NCMN*-O-*demethylation in liver microsomes from different species.

Enzyme source	*V_m1_*	*V_m2_*	*K_m1_*	*K_m2_*	*V_m_/K_m_*
HLM	25.61 ± 0.15		9.54 ± 0.24		2684.48
CyLM	8.43 ± 0.91	0.28 ± 0.08	198.70 ± 39.66	0.18 ± 0.09	1559.45 ^1^
DLM	13.53 ± 0.34		61.79 ± 3.52		218.96
PLM	28.22 ± 5.82	3.35 ± 1.20	242.80 ± 111.80	5.70 ± 3.27	587.44 ^1^
MLM	33.17 ± 0.42		22.80 ± 1.22		1454.82
RLM	78.42 ± 31.17	5.71 ± 0.45	1134.00 ± 482.10	5.66 ± 0.80	1007.94 ^1^

*K_m_* values are in µM; *V_m_* values are in nmol/min/mg for liver microsomes; *V_m_/K_m_* values are in ml/min/mg for liver microsomes. The range of substrate concentrations was 1–150 µM. ^1^ *V_m_/K_m_* values were calculated by using the lower V_m_ and *K_m_* values.

## Supplementary Materials

The following are available online, Figure S1: LC-ESI-MS spectra of NCMN and NCHN, Figure S2: Michaelis-Menten plot of NCMN*-O-*demethylation in rat CYP1A2. Figure S3: Phylogenic tree of CYP1A1 and CYP1A2, Table S1: Kinetic parameters of NCMN*-O-*demethylation determined in different enzyme sources. Table S2: Percent identity matrix of similarity of amino acid sequences of CYP1A1 from different species, Table S3: Percent identity matrix of similarity of amino acid sequences of CYP1A2 from different species. Please mention supplementary materials in main text at least once. (We have mentioned supplementary materials in main text, please see the details in the revised main text).

## References

[1] Zanger U.M., Schwab M. (2013). Cytochrome P450 enzymes in drug metabolism: Regulation of gene expression, enzyme activities, and impact of genetic variation. Pharmacol. Ther..

[2] Guengerich F.P. (2004). Cytochrome P450: What have we learned and what are the future issues?. Drug Metab. Rev..

[3] Kim D., Guengerich F.P. (2004). Selection of human cytochrome P450 1A2 mutants with enhanced catalytic activity for heterocyclic amine N-hydroxylation. Biochemistry.

[4] Kim D., Guengerich F.P. (2005). Cytochrome P450 activation of arylamines and heterocyclic amines. Annu. Rev. Pharmacol. Toxicol..

[5] Ma Q., Lu A.Y. (2007). CYP1A induction and human risk assessment: An evolving tale of in vitro and in vivo studies. Drug Metab. Dispos..

[6] Michaud V., Frappier M., Dumas M.C., Turgeon J. (2010). Metabolic activity and mRNA levels of human cardiac CYP450s involved in drug metabolism. PLoS ONE.

[7] Ohtsuki S., Schaefer O., Kawakami H., Inoue T., Liehner S., Saito A., Ishiguro N., Kishimoto W., Ludwig-Schwellinger E., Ebner T. (2012). Simultaneous absolute protein quantification of transporters, cytochromes P450, and UDP-glucuronosyltransferases as a novel approach for the characterization of individual human liver: Comparison with mRNA levels and activities. Drug Metab. Dispos..

[8] Kawakami H., Ohtsuki S., Kamiie J., Suzuki T., Abe T., Terasaki T. (2011). Simultaneous absolute quantification of 11 cytochrome P450 isoforms in human liver microsomes by liquid chromatography tandem mass spectrometry with in silico target peptide selection. J. Pharm. Sci..

[9] Proctor R.N. (2001). Tobacco and the global lung cancer epidemic. Nat. Rev. Cancer.

[10] Dobrinas M., Cornuz J., Oneda B., Kohler S.M., Puhl M., Eap C.B. (2011). Impact of smoking, smoking cessation, and genetic polymorphisms on CYP1A2 activity and inducibility. Clin. Pharmacaol. Harmacol. Ther..

[11] Wang H., Zhang Z., Han S., Lu Y., Feng F., Yuan J. (2012). CYP1A2 rs762551 polymorphism contributes to cancer susceptibility: A meta-analysis from 19 case-control studies. BMC Cancer.

[12] Sergentanis T.N., Economopoulos K.P. (2010). Four polymorphisms in cytochrome P450 1A1 (CYP1A1) gene and breast cancer risk: A meta-analysis. Breast Cancer Res. Treat..

[13] Berthou F., Guillois B., Riche C., Dreano Y., Jacqz-Aigrain E., Beaune P.H. (1992). Interspecies variations in caffeine metabolism related to cytochrome P4501A enzymes. Xenobiotica.

[14] Martignoni M., Groothuis G.M., de Kanter R. (2006). Species differences between mouse, rat, dog, monkey and human CYP-mediated drug metabolism, inhibition and induction. Expert Opin. Drug. Metab. Toxicol..

[15] Dai Z., Feng L., Jin Q., Cheng H., Li Y., Ning J., Yu Y., Ge G., Cui J., Yang L. (2017). A practical strategy to design and develop an isoform-specific fluorescent probe for a target enzyme: CYP1A1 as a case study. Chem. Sci..

[16] Zhang H., Fan J., Wang J., Zhang S., Dou B., Peng X. (2013). An off-on COX-2-specific fluorescent probe: Targeting the Golgi apparatus of cancer cells. J. Am. Chem. Soc..

[17] Jin Q., Feng L., Wang D.D., Dai Z.R., Wang P., Zou L.W., Liu Z.H., Wang J.Y., Yu Y., Ge G.B. (2015). A Two-Photon Ratiometric Fluorescent Probe for Imaging Carboxylesterase 2 in Living Cells and Tissues. ACS Appl. Mater. Interfaces.

[18] Shimada T., Yamazaki H., Mimura M., Wakamiya N., Ueng Y.F., Guengerich F.P., Inui Y. (1996). Characterization of microsomal cytochrome P450 enzymes involved in the oxidation of xenobiotic chemicals in human fetal liver and adult lungs. Drug Metab. Dispos..

[19] Smith G.B., Harper P.A., Wong J.M., Lam M.S., Reid K.R., Petsikas D., Massey T.E. (2001). Human lung microsomal cytochrome P4501A1 (CYP1A1) activities: Impact of smoking status and CYP1A1, aryl hydrocarbon receptor, and glutathione S-transferase M1 genetic polymorphisms. Cancer Epidemiol. Prev. Biomark..

[20] Sípal Z., Ahlenius T., Bergstrand A., Rodriquez L., Jakobsson S.W. (1979). Oxidative biotransformation of benzo(a)pyrene by human lung microsomal fractions prepared from surgical specimens. Xenobiotica.

[21] Devereux T.R., Massey T.E., Van Scott M.R., Yankaskas J., Fouts J.R. (1986). Xenobiotic metabolism in human alveolar type II cells isolated by centrifugal elutriation and density gradient centrifugation. Cancer Res..

[22] Dai Z., Ge G., Feng L., Ning J., Hu L., Jin Q., Wang D., Lv X., Dou T., Cui J. (2015). A Highly Selective Ratiometric Two-Photon Fluorescent Probe for Human Cytochrome P450 1A. J. Am. Chem. Soc..

[23] Fuhr U., Jetter A., Kirchheiner J. (2007). Appropriate phenotyping procedures for drug metabolizing enzymes and transporters in humans and their simultaneous use in the "cocktail" approach. Clin. Pharmacol. Ther..

[24] Zhou S.F., Wang B., Yang L.P., Liu J.P. (2010). Structure, function, regulation and polymorphism and the clinical significance of human cytochrome P450 1A2. Drug Metab. Rev..

[25] Wogan G.N., Hecht S.S., Felton J.S., Conney A.H., Loeb L.A. (2004). Environmental and chemical carcinogenesis. Semin. Cancer Biol..

[26] Shimada T., Fujii-Kuriyama Y. (2004). Metabolic activation of polycyclic aromatic hydrocarbons to carcinogens by cytochromes P450 1A1 and 1B1. Cancer Sci..

[27] Cornelis M.C., El-Sohemy A., Kabagambe E.K., Campos H. (2006). Coffee, CYP1A2 genotype, and risk of myocardial infarction. JAMA.

[28] Androutsopoulos V.P., Tsatsakis A.M., Spandidos D.A. (2009). Cytochrome P450 CYP1A1: Wider roles in cancer progression and prevention. BMC Cancer.

[29] Sharer J.E., Shipley L.A., Vandenbranden M.R., Binkley S.N., Wrighton S.A. (1995). Comparisons of Phase I and Phase II in vitro hepatic enzyme activities of human, dog, rhesus monkey, and cynomolgus monkey. Drug Metab. Dispos..

[30] Santostefano M.J., Ross D.G., Savas U., Jefcoate C.R., Birnbaum L.S. (1997). Differential time-course and dose-response relationships of TCDD-induced CYP1B1, CYP1A1, and CYP1A2 proteins in rats. Biochem. Biophys. Res. Commun..

[31] Choudhary D., Jansson I., Schenkman J.B., Sarfarazi M., Stoilov I. (2003). Comparative expression profiling of 40 mouse cytochrome P450 genes in embryonic and adult tissues. Arch. Biochem. Biophys..

[32] Graham R.A., Downey A., Mudra D., Krueger L., Carroll K., Chengelis C., Madan A., Parkinson A. (2002). In Vivo and in vitro induction of cytochrome P450 enzymes in beagle dogs. Drug Metab. Dispos..

[33] Zuber R., Anzenbacherová E., Anzenbacher P. (2002). Cytochromes P450 and experimental models of drug metabolism. J. Cell. Mol. Med..

[34] Karthikeyan B.S., Suvaithenamudhan S., Akbarsha M.A., Parthasarathy S. (2018). Analysis of species-selectivity of human, mouse and rat Cytochrome P450 1A and 2B subfamily enzymes using molecular modeling, docking and dynamics simulations. Cell. Biochem. Biophys..

[35] Rendic S., Guengerich F.P. (2012). Contributions of Human Enzymes in Carcinogen Metabolism. Chem. Res. Toxicol..

[36] Sridhar J., Liu J., Foroozesh M., Klein Stevens C.L. (2012). Inhibition of Cytochrome P450 Enzymes by Quinones and Anthraquinones. Chem. Res. Toxicol..

[37] Liu J., Taylor S.F., Dupart P.S., Arnold C.L., Sridhar J., Jiang Q., Wang Y., Skripnikova E.V., Zhao M., Foroozesh M. (2013). Pyranoflavones: A group of small-molecule probes for exploring the active site cavities of cytochrome P450 enzymes 1A1, 1A2, and 1B1. J. Med. Chem..

[38] Liu J., Sridhar J., Foroozesh M. (2013). Cytochrome P450 family 1 inhibitors and structure-activity relationships. Molecules.

[39] Zhou Q., Yan X.F., Zhang Z.M., Pan W.S., Zeng S. (2007). Rational prescription of drugs within similar therapeutic or structural class for gastrointestinal disease treatment: Drug metabolism and its related interactions. World J. Gastroenterol..

[40] Toon S., Hopkins K.J., Garstang F.M., Aarons L., Sedman A., Rowland M. (1987). Enoxacin-warfarin interaction: Pharmacokinetic and stereochemical aspects. Clin. Pharmacol. Ther..

[41] Taura K., Naito E., Ishii Y., Mori M., Oguri K., Yamada H., Medicine S.O., Sciences A.S.O.P., Sciences C.O.P., University K. (2004). Cytochrome P450 1A1 (CYP1A1) inhibitor alpha-naphthoflavone interferes with UDP-glucuronosyltransferase (UGT) activity in intact but not in permeabilized hepatic microsomes from 3-methylcholanthrene-treated rats: Possible involvement of UGT-P450 interactions. Biol. Pharm. Bull..

[42] Bogaards J.J.P., Bertrand M., Jackson P., Oudshoorn M.J., Weaver R.J., Van Bladeren P.J., Walther B. (2000). Determining the best animal model for human cytochrome P450 activities: A comparison of mouse, rat, rabbit, dog, micropig, monkey and man. Xenobiotica.

[43] Walsh A.A., Szklarz. G.D., Scott E.E. (2013). Human Cytochrome P450 1A1 Structure and Utility in Understanding Drug. J. Biol. Chem..

[44] Balani S.K., Zhu T., Yang T.J., Liu Z., He B., Lee F.W. (2002). Effective dosing regimen of 1-aminobenzotriazole for inhibition of antipyrine clearance in rats, dogs, and monkeys. Drug Metab. Dispos..

[45] Bjornsson T.D., Callaghan J.T., Einolf H.J., Fischer V., Gan L., Grimm S., Kao J., King S.P., Miwa G., Ni L. (2003). The conduct of in vitro and in vivo drug-drug interaction studies: A PhRMA perspective. J. Clin. Pharmacol..

[46] Tassaneeyakul W., Birkett D.J., Veronese M.E., McManus M.E., Tukey R.H., Miners J.O. (1994). Direct characterization of the selectivity of furafylline as an inhibitor of human cytochromes P450 1A1 and 1A2. Pharmacogenetics.

[47] Baldwin S.J., Bloomer J.C., Smith G.J., Ayrton A.D., Clarke S.E., Chenery R.J. (1995). Ketoconazole and sulphaphenazole as the respective selective inhibitors of P4503A and 2C9. Xenobiotica.

