# Supplementary material for: Interspecies Variation in NCMN-O-Demethylation in Liver Microsomes from Various Species
Source: Molecules. 2019 Jul 30;24(15):2765. doi: 10.3390/molecules24152765 (PMC6695839; doi:10.3390/molecules24152765)
Supplement: Supplementary file 1 [file molecules-24-02765-s001.pdf]

SUPPLEMENTARY MATERIAL

Interspecies variation in NCMN-O-demethylation in liver microsomes from various species

Zi-Ru Dai [1,†], Gui-Bo Sun [1,†], Jia-Da Yang [2,†], Jie Hou [3], Ping Zhou [1], Wei-Jie Xie [1], Guang-Bo Ge [4,*], Xiao-Bo Sun [1, *], and Ling Yang [4]

[1] Key Laboratory of Bioactive Substances and Resources Utilization of Chinese Herbal Medicine, Ministry of Education, Institute of Medicinal Plant Development, Peking Union Medical College and Chinese Academy of Medical Sciences, Beijing 100193, China; athenadai219@163.com (Z.D.); sunguibo@126.com (G.S.); zhoup0520@163.com (P.Z.); xwjginseng@126.com (W.X.)

[2] School of Environment and Life Science, Kaili University, Kaili 556011, China; yangjiada2@163.com (J.Y.);

[3] College of Basic Medical Sciences, Dalian Medical University, Dalian 116044, China; houjie@dlmedu.edu.cn (J.H.);

[4] Institute of Interdisciplinary Integrative Medicine Research, Shanghai University of Traditional Chinese Medicine, Shanghai 201203, China; yling@dicp.ac.cn (L.Y.)

* Correspondence: geguangbo@dicp.ac.cn (G.G.); sun_xiaobo163@163.com (X.S.);

Tel.:+86-21-5132-3184 Tel.:+86-10-5783-3013

† These authors contributed equally to this work.

Table S1. Kinetic parameters of NCMN-O-demethylation determined in different enzyme sources.

Enzyme sources	V_{max}	K_m(μM)	V_{max}/ K_m
HLM	25.61 ± 0.15	9.54 ± 0.24	2684.48
CYP1A2 (human)	32.06 ± 0.50	9.80 ± 0.67	3271.43
CYP1A2 (Rat)	7.48±0.05	4.04±0.16	1851.48

K_m values are in μM; V_{max} values are in nmol/min/mg for liver microsomes; V_{max}/K_m values are in ml/min/mg for liver microsomes, or in nmol/min/nmol CYP for CYP1A2. The range of substrate concentrations was 1–150 μM.

Table S2. Percent Identity Matrix of similarity of amino acid sequences of Cytochrome P450 1A1 from different species. (Created by Clustal 2.1)

**Species**	**rat**	**mouse**	**dog**	**human**	**minipig**	**monkey**
human	79.60	80.39	82.08	100.00	82.35	94.33
rat	100.00	93.12	76.92	79.80	79.45	79.60
mouse	93.12	100.00	77.69	80.39	78.48	80.43
dog	76.92	77.69	100.00	82.08	83.14	82.45
minipig	79.45	78.48	83.14	82.35	100.00	83.46

monkey	79.60	80.43	82.45	94.14	83.46	100.00

Table S3. Percent Identity Matrix of similarity of amino acid sequences of Cytochrome P450 1A2 from different species. (Created by Clustal 2.1)

**Species**	**rat**	**mouse**	**dog**	**human**	**minipig**	**monkey**
human	83.09	82.76	81.78	100.00	81.39	93.02
rat	100.00	93.37	72.32	83.09	75.24	76.07
mouse	93.37	100.00	70.95	82.76	76.23	74.12
dog	73.29	71.93	100.00	81.78	78.26	81.39
minipig	73.32	73.55	78.26	81.39	100.00	82.17
monkey	76.07	74.12	81.20	93.02	77.43	100.00

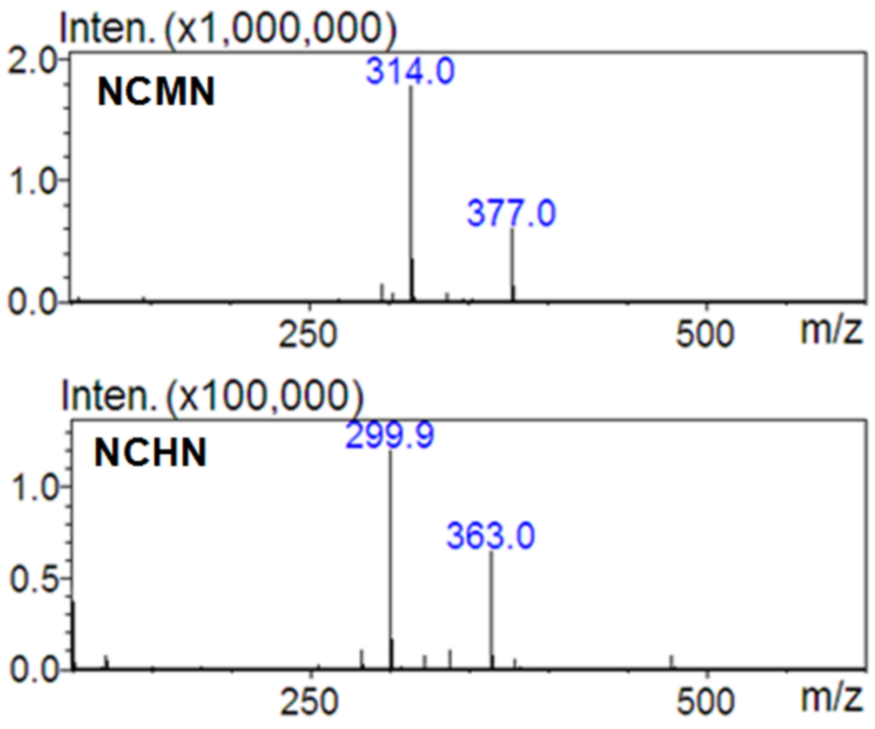

Figure S1. The LC-ESI-MS spectra of NCMN and NCHN under positive ion mode.

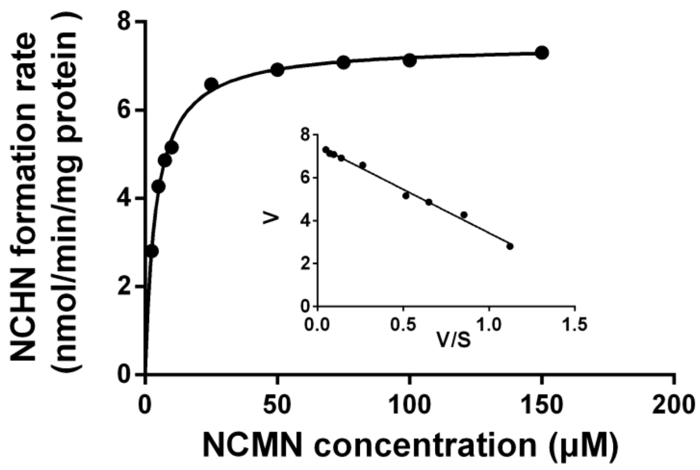

Figure S2. Michaelis-Menten plot of NCMN-O-demethylation in rat CYP1A2.

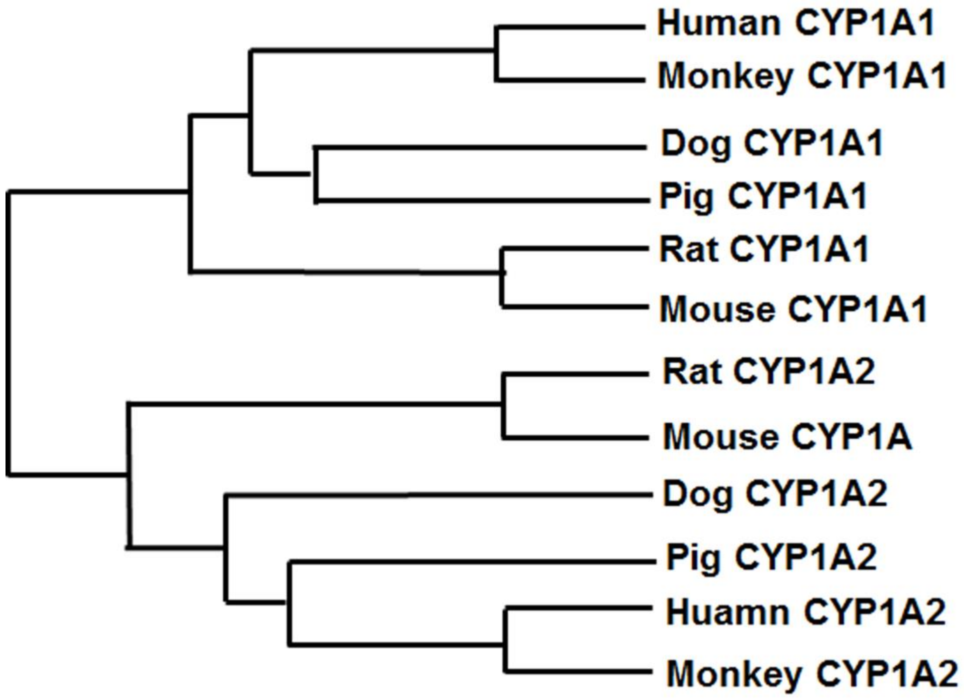

Figure S3. Phylogenic tree of CYP1A1 and CYP1A2 from different species. (created by MEGA7.0)

*These species amino acid sequences were inquired from website Ensembl (http://asia.ensembl.org/index.html) and NCBI (https://www.ncbi.nlm.nih.gov/protein/)

Human CYP1A1：NP_000490.1 [Homo sapiens]

Human CYP1A2：NP_000752.2 [Homo sapiens]

Monkey CYP1A1：NP_001035328.1 [Macaca fascicularis]

Monkey CYP1A2：NP_001306412.1 [Macaca fascicularis]

Dog CYP1A1：XP_003433938.1 [Canis lupus familiaris]

Dog CYP1A2：NP_001008720.1 [Canis lupus familiaris]

Pig CYP1A1：NP_999577.1 [Sus scrofa]

Pig CYP1A2：NP_001153086.1 [Sus scrofa]

Rat CYP1A1：NP_036672.2 [Rattus norvegicus]

Rat CYP1A2：NP_036673.3 [Rattus norvegicus]

Mouse CYP1A1：NP_034122.1 [Mus musculus]

Mouse CYP1A2：NP_034123.1 [Mus musculus]
